# The complete chloroplast genome of copper-tolerance plant *Elsholtzia splendens*

**DOI:** 10.1080/23802359.2019.1644241

**Published:** 2019-07-22

**Authors:** Lele Ding, Xiaomeng Zhao, Long Su, Hongyun Peng, Cheng Sun

**Affiliations:** a Ministry of Education Key Laboratory of Environment Remediation and Ecological Health, College of Environmental and Resource Sciences, Zhejiang University, Hangzhou, China;; b Institute of Apicultural Research, Chinese Academy of Agricultural Sciences, Beijing, China

**Keywords:** *Elsholtzia splendens*, copper-tolerance plant, chloroplast genome

## Abstract

*Elsholtzia splendens* is a copper-tolerance plant colonized in copper mines in southern China. In this study, we sequenced and *de novo* assembled the complete chloroplast genome of *E. splendens*. The complete chloroplast genome is 150,761 bp (37.8% of GC) in length and contains 87 protein-coding genes, 38 tRNA genes, and 8 rRNA genes. Phylogenetic analysis revealed that among the 11 Lamiaceae species, *Perilla citriodor* is the closest relative of *E. splendens*. The complete chloroplast genome of *E. splendens* provides a valuable resource for comparative and evolutionary analysis among Lamiaceae species and may be helpful in understanding the molecular mechanism of copper tolerance in *E. splendens*.

*Elsholtzia splendens* is a plant species in colonized copper mines in southern China (Xie and Xu 1952), which was known as ‘copper grass’ for its crucial role in phytoremediation of soils contaminated with copper. Researches have been carried out to elucidate its role in remediating copper-contaminated soils (Li et al. 2003; Liu et al. 2014; Zhao et al. 2016) and studies also have suggested that its chloroplast is implicated in copper stress (Teisseire et al. 1999; Peng et al. 2014). However, to date, little information is available on the genetic make of *E. splendens* chloroplast.

The leaves of *E. splendens* were collected from a copper-rich site located in Zhuji county, Zhejiang province, China (N29°36′44″; E120°22′37″), with some specimen being stored in Zhejiang University Museum (Accession#: Esplendens-001). The total DNA was extracted by CTAB method (Doyle and Doyle 1990). DNA sequencing library was prepared using standard Illumina protocols. The constructed library was sequenced with Illumina HiSeq2000 platform and paired-end reads were produced, with a read length of 150 bp. The raw sequencing reads have been deposited in NCBI SRA (Accession #: PRJNA545842).

To obtain the complete chloroplast genome sequence of *E. splendens*, firstly, the shotgun sequences of *E. splendens* was assembled using SPAdes software (Bankevich et al. 2012). Secondly, the complete chloroplast genome of *Sesamum indicum* (Eguiluz et al. 2017) was used as a query sequence to do BLASTn searches against the assembled shotgun sequences of *E. splendens*, through which we could get fragments of *E. splendens* chloroplast genome. Finally, the obtained fragment sequences were used as seeds of NOVOPlasty software (Dierckxsens et al. 2017) to perform *de novo* assembly of *E. splendens* chloroplast genome. Chloroplast genes were annotated using DOGMA program (Wyman et al. 2004) and CpGAVAS pipeline (Liu et al. 2012). Multiple sequence alignment was done by MAFFT (Nakamura et al. 2018), with manually edited using BioEdit (Hall 1999). Phylogenetic analysis was performed among 11 Lamiaceae species, with *Arabidopsis thaliana* (GenBank: NC 000932.1) serving as outgroup. The phylogenetic tree was constructed by neighbour-joining method with 1000 bootstrap replicates using MEGA7 (Kumar et al. 2016).

The complete chloroplast genome sequence of *E. Splendens* (GenBank: MH700782) is 150,761 bp in length (37.8% of GC). The chloroplast genome has an LSC region of 82,144 bp, an SSC region of 17,473 bp, and a pair of inverted repeats (IRa and IRb) of 25,572 bp. The chloroplast genome of *E. splendens* encodes 133 genes, including 87 protein-encoding genes, 38 tRNA genes, and 8 rRNA genes. There are three protein-coding genes (*ycf3*, *rps12*, and *clpP*) with 3 exons, 8 protein-coding genes with 2 exons (*atpF*, *ndhA*, *ndhB*, *petB*, *rpl16*, *rpl2*, *rpoC1*, and *rps16*), and all other genes with 1 exon. Phylogenetic analysis among the 11 Lamiaceae species (Figure 1) indicates that *Perilla citriodor* is the closest relative of *E. splendens*. The complete chloroplast genome of *E. splendens* provides a valuable resource for comparative and evolutionary analysis of species within Lamiaceae and might be helpful in understanding the molecular mechanism of copper tolerance in *E. splendens*.

**Figure 1.**
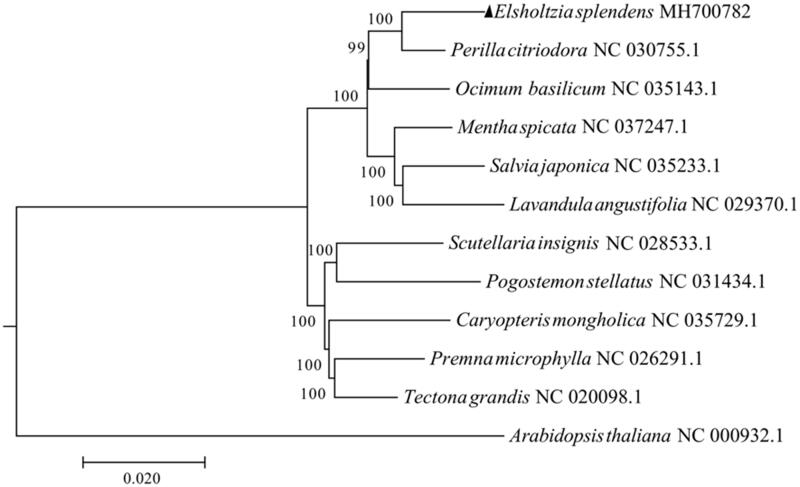
Phylogenetic analysis of 11 chloroplast genome sequences of Lamiaceae species, with the use of chloroplast genome sequence from *Arabidopsis thaliana* as outgroup. Black triangle indicates the focal chloroplast genome of this study. Numbers beside each node are percentages of 1000 bootstrap values. GenBank accession numbers were followed after their corresponding species names.
